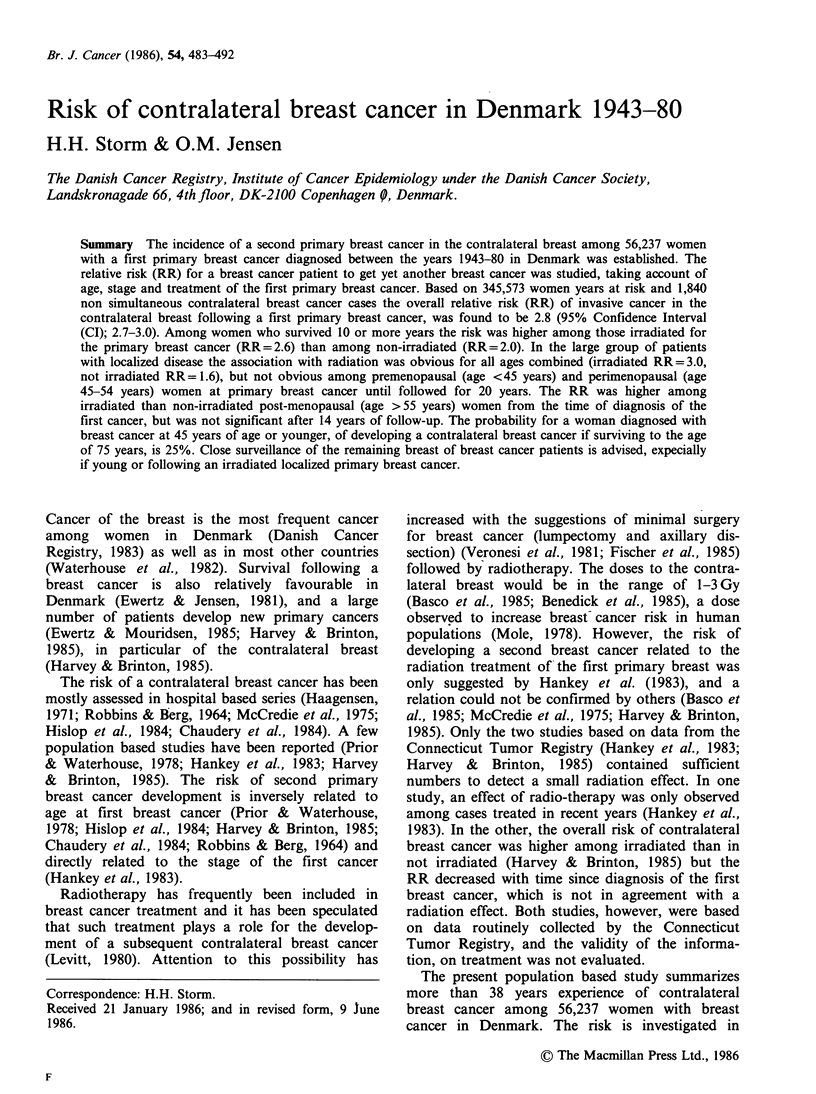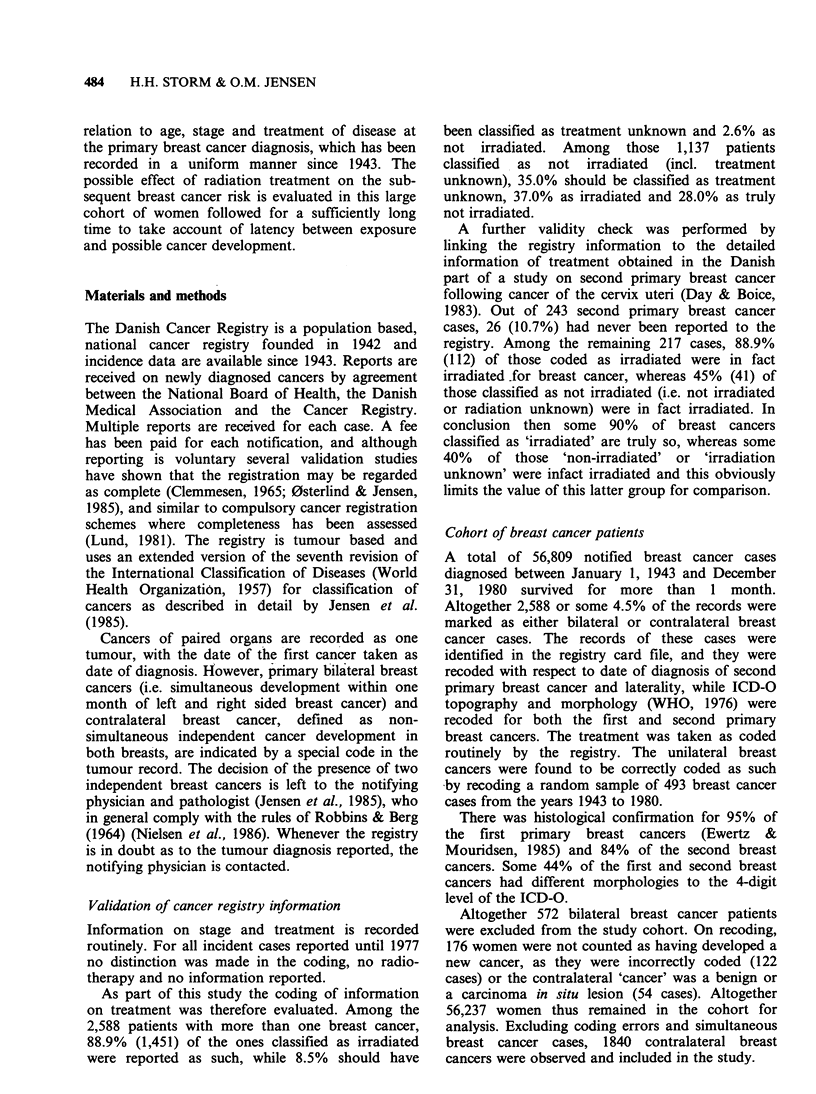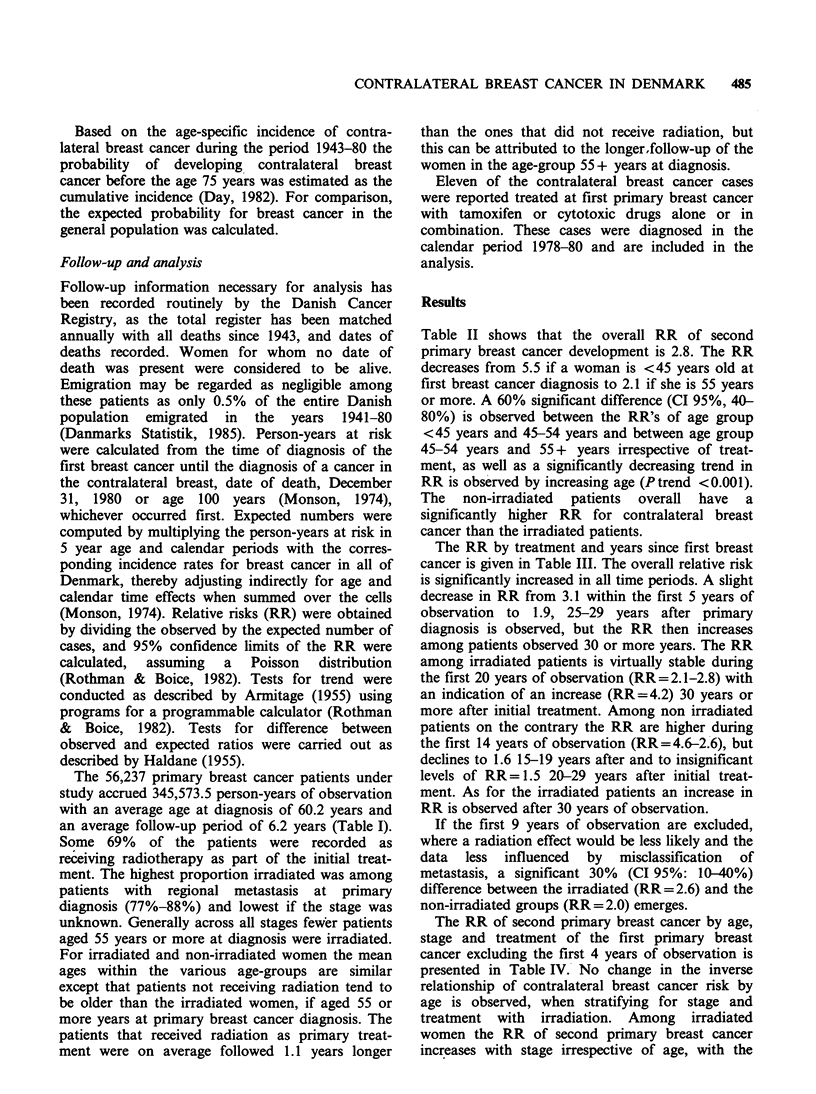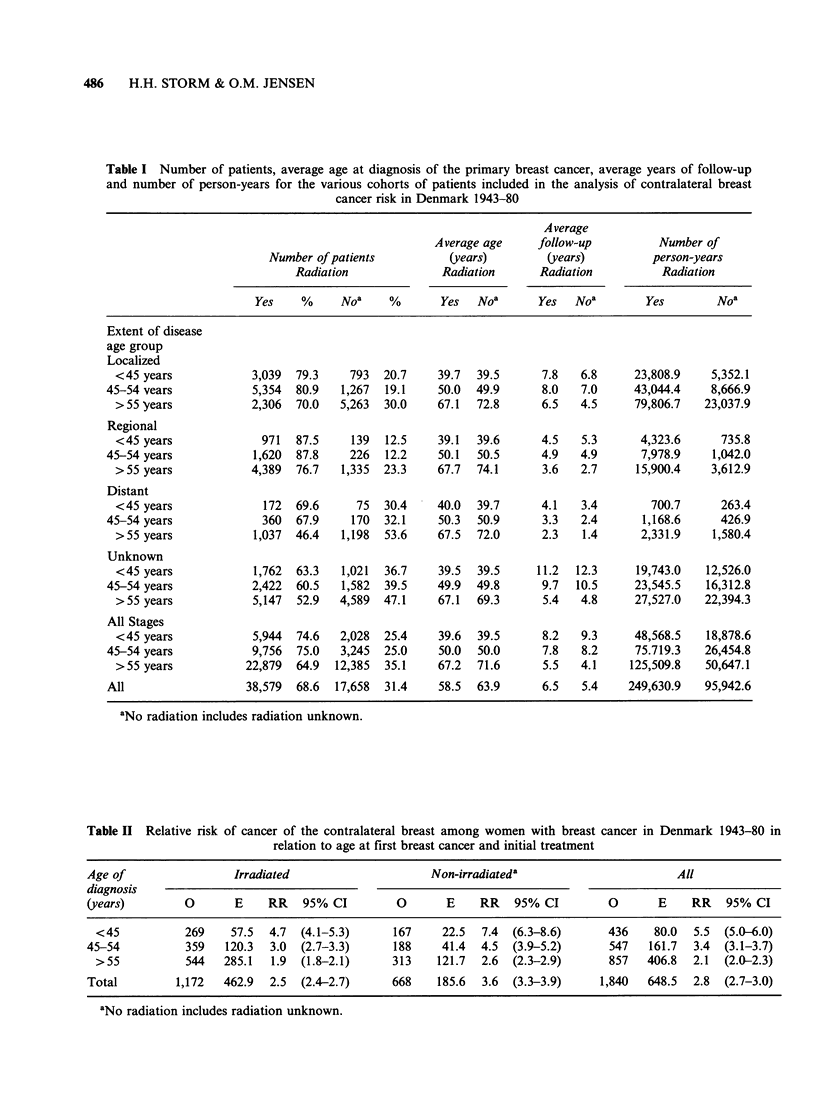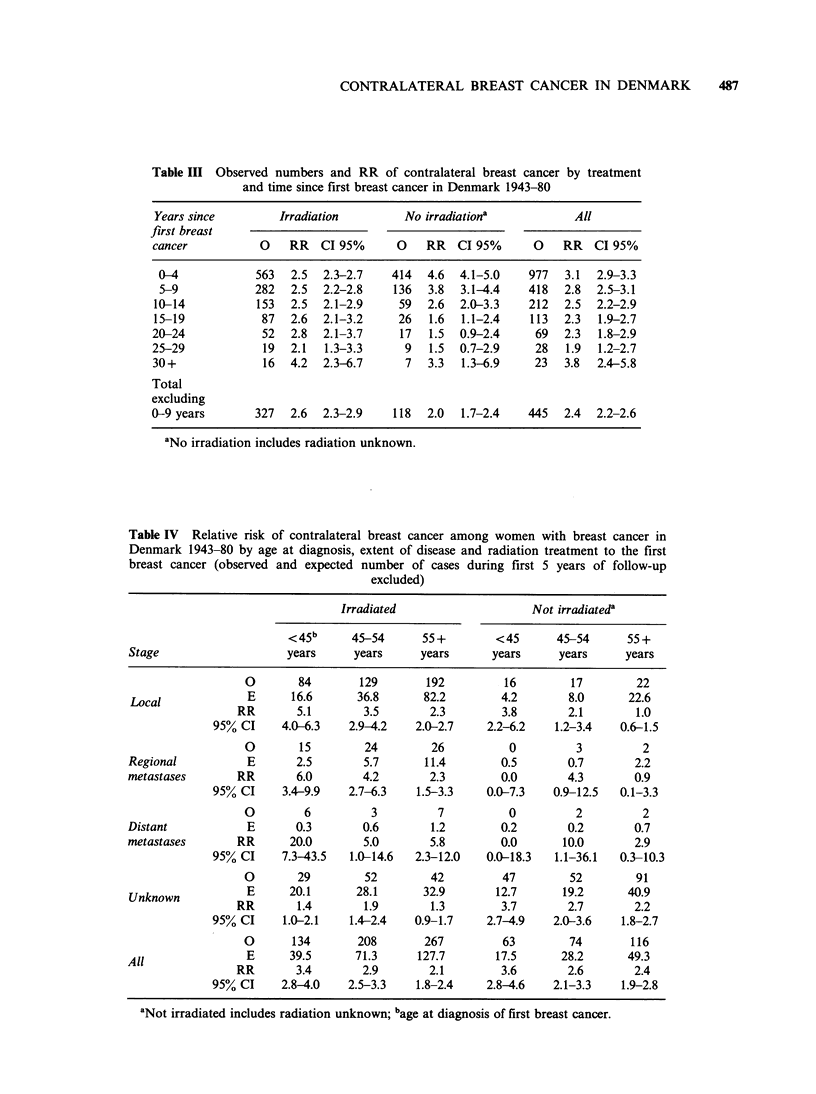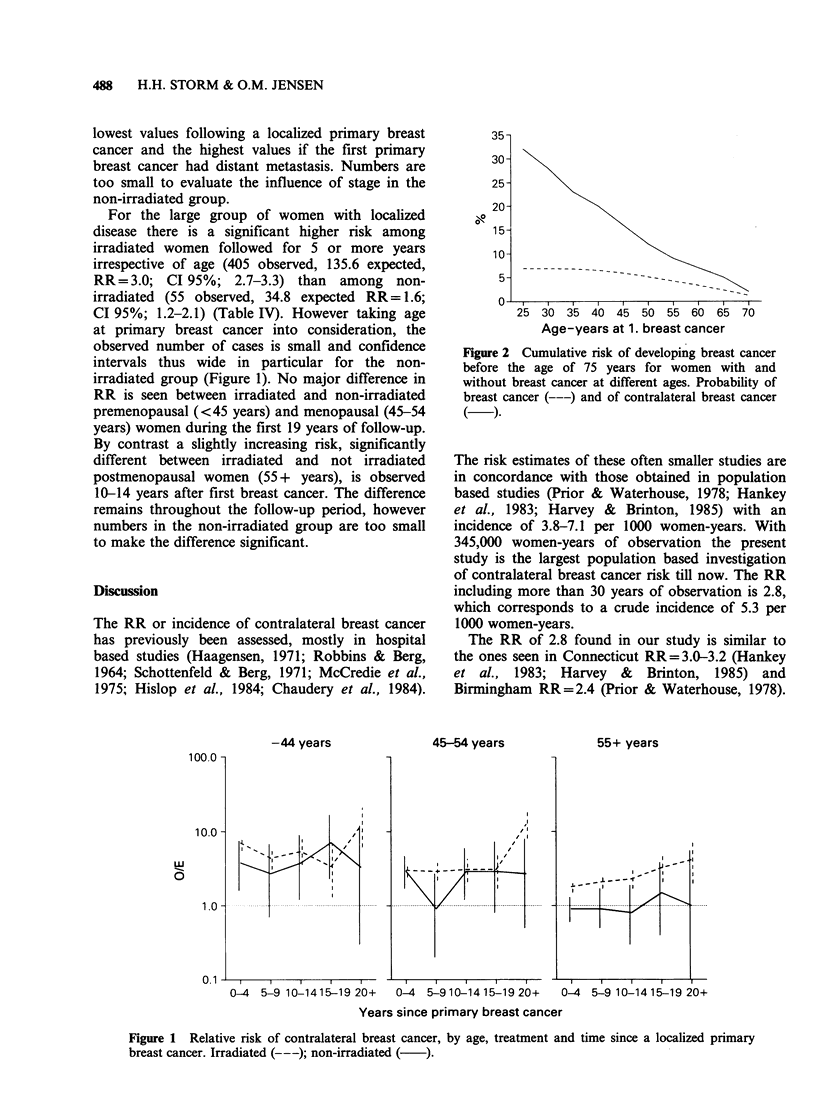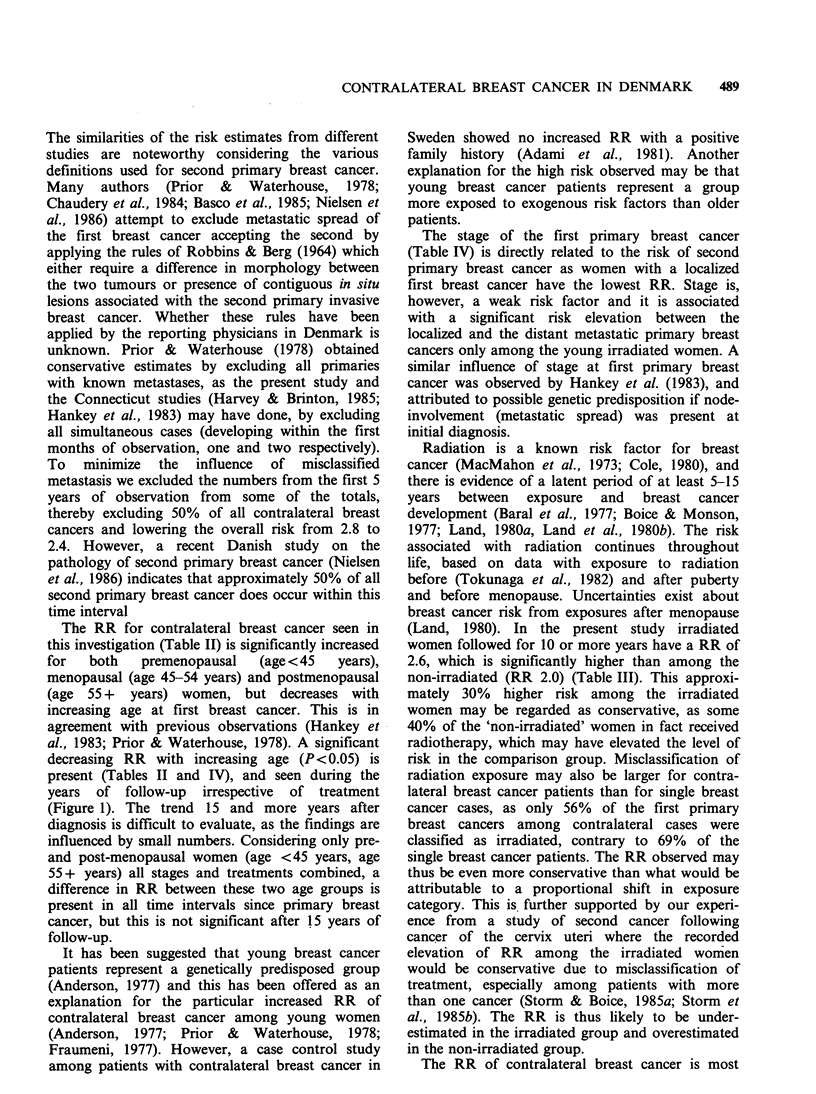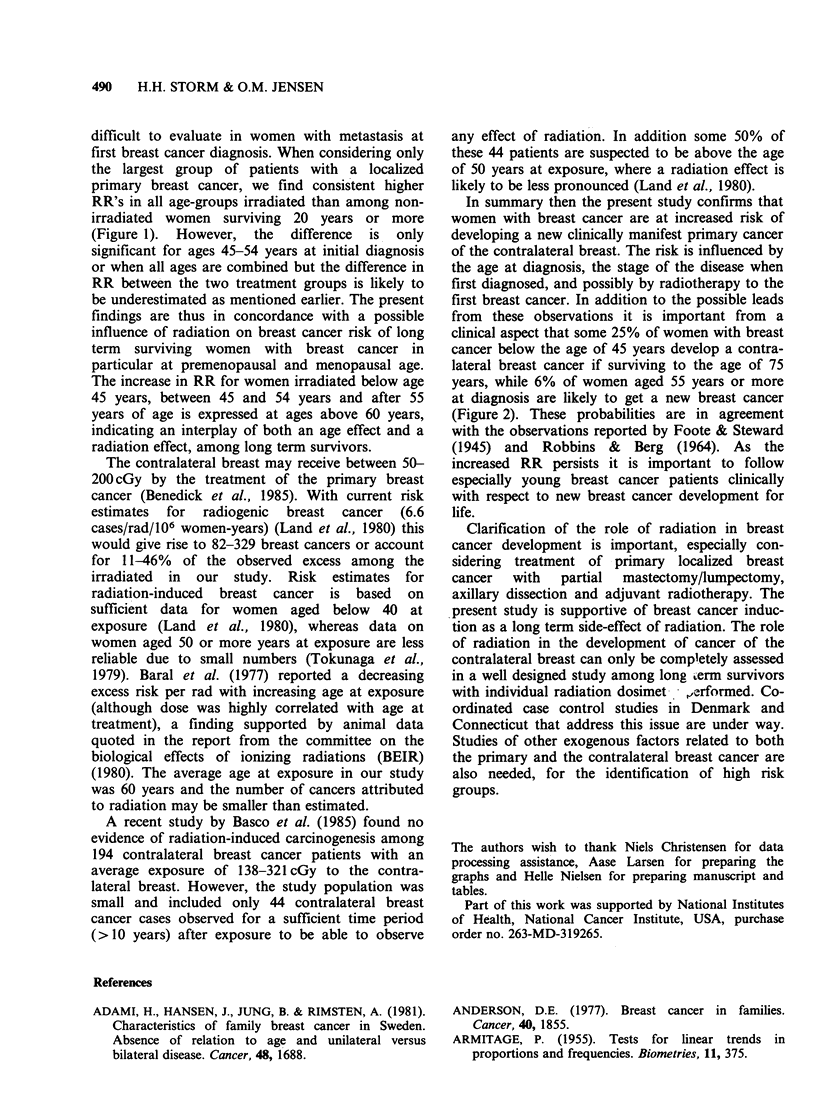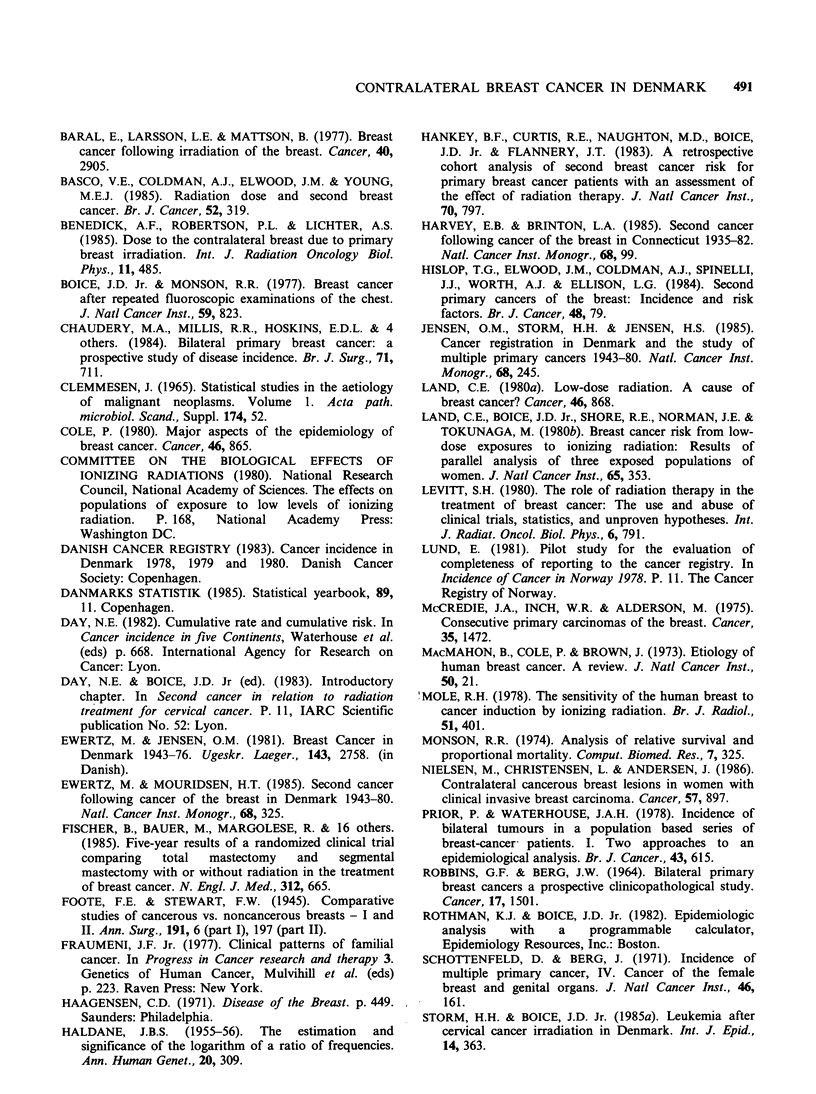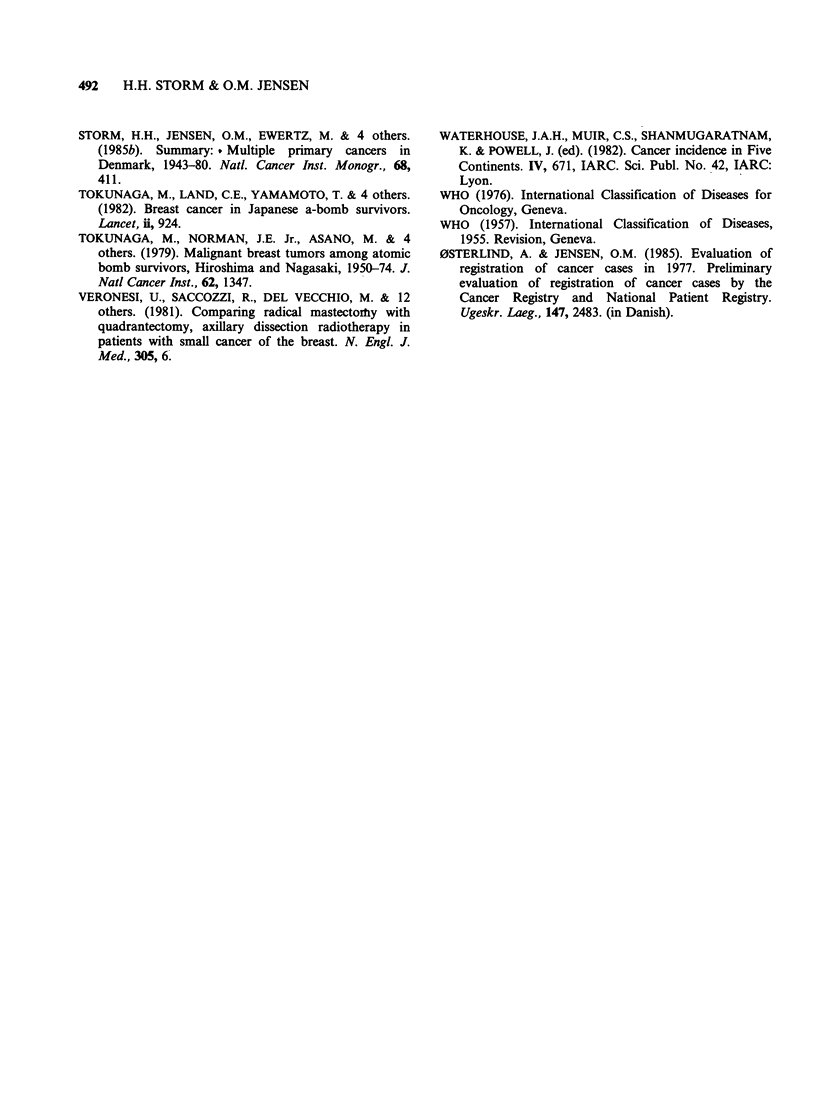# Risk of contralateral breast cancer in Denmark 1943-80.

**DOI:** 10.1038/bjc.1986.201

**Published:** 1986-09

**Authors:** H. H. Storm, O. M. Jensen

## Abstract

The incidence of a second primary breast cancer in the contralateral breast among 56,237 women with a first primary breast cancer diagnosed between the years 1943-80 in Denmark was established. The relative risk (RR) for a breast cancer patient to get yet another breast cancer was studied, taking account of age, stage and treatment of the first primary breast cancer. Based on 345,573 women years at risk and 1,840 non simultaneous contralateral breast cancer cases the overall relative risk (RR) of invasive cancer in the contralateral breast following a first primary breast cancer, was found to be 2.8 (95% Confidence Interval (CI); 2.7-3.0). Among women who survived 10 or more years the risk was higher among those irradiated for the primary breast cancer (RR = 2.6) than among non-irradiated (RR = 2.0). In the large group of patients with localized disease the association with radiation was obvious for all ages combined (irradiated RR = 3.0, not irradiated RR = 1.6), but not obvious among premenopausal (age less than 45 years) and perimenopausal (age 45-54 years) women at primary breast cancer until followed for 20 years. The RR was higher among irradiated than non-irradiated post-menopausal (age greater than 55 years) women from the time of diagnosis of the first cancer, but was not significant after 14 years of follow-up. The probability for a woman diagnosed with breast cancer at 45 years of age or younger, of developing a contralateral breast cancer if surviving to the age of 75 years, is 25%. Close surveillance of the remaining breast of breast cancer patients is advised, especially if young or following an irradiated localized primary breast cancer.


*Br. J. Cancer* (1986), **54**, 483–492

### Risk of contralateral breast cancer in Denmark 1943–80

H.H. Storm & O.M. Jensen

*The Danish Cancer Registry, Institute of Cancer Epidemiology under the Danish Cancer Society, Landskronagade 66, 4th floor, DK-2100 Copenhagen Ø, Denmark.*

**Summary** The incidence of a second primary breast cancer in the contralateral breast among 56,237 women with a first primary breast cancer diagnosed between the years 1943–80 in Denmark was established. The relative risk (RR) for a breast cancer patient to get yet another breast cancer was studied, taking account of age, stage and treatment of the first primary breast cancer. Based on 345,573 women years at risk and 1,840 non simultaneous contralateral breast cancer cases the overall relative risk (RR) of invasive cancer in the contralateral breast following a first primary breast cancer, was found to be 2.8 (95% Confidence Interval (CI); 2.7–3.0). Among women who survived 10 or more years the risk was higher among those irradiated for the primary breast cancer (RR=2.6) than among non-irradiated (RR=2.0). In the large group of patients with localized disease the association with radiation was obvious for all ages combined (irradiated RR=3.0, not irradiated RR=1.6), but not obvious among premenopausal (age <45 years) and perimenopausal (age 45–54 years) women at primary breast cancer until followed for 20 years. The RR was higher among irradiated than non-irradiated post-menopausal (age >55 years) women from the time of diagnosis of the first cancer, but was not significant after 14 years of follow-up. The probability for a woman diagnosed with breast cancer at 45 years of age or younger, of developing a contralateral breast cancer if surviving to the age of 75 years, is 25%. Close surveillance of the remaining breast of breast cancer patients is advised, expecially if young or following an irradiated localized primary breast cancer.

Cancer of the breast is the most frequent cancer among women in Denmark (Danish Cancer Registry, 1983) as well as in most other countries (Waterhouse *et al.*, 1982). Survival following a breast cancer is also relatively favourable in Denmark (Ewertz & Jensen, 1981), and a large number of patients develop new primary cancers (Ewertz & Mouridsen, 1985; Harvey & Brinton, 1985), in particular of the contralateral breast (Harvey & Brinton, 1985).

The risk of a contralateral breast cancer has been mostly assessed in hospital based series (Haagensen, 1971; Robbins & Berg, 1964; McCredie *et al.*, 1975; Hislop *et al.*, 1984; Chaudery *et al.*, 1984). A few population based studies have been reported (Prior & Waterhouse, 1978; Hankey *et al.*, 1983; Harvey & Brinton, 1985). The risk of second primary breast cancer development is inversely related to age at first breast cancer (Prior & Waterhouse, 1978; Hislop *et al.*, 1984; Harvey & Brinton, 1985; Chaudery *et al.*, 1984; Robbins & Berg, 1964) and directly related to the stage of the first cancer (Hankey *et al.*, 1983).

Radiotherapy has frequently been included in breast cancer treatment and it has been speculated that such treatment plays a role for the development of a subsequent contralateral breast cancer (Levitt, 1980). Attention to this possibility has increased with the suggestions of minimal surgery for breast cancer (lumpectomy and axillary dissection) (Veronesi *et al.*, 1981; Fischer *et al.*, 1985) followed by radiotherapy. The doses to the contralateral breast would be in the range of 1–3 Gy (Basco *et al.*, 1985; Benedick *et al.*, 1985), a dose observed to increase breast cancer risk in human populations (Mole, 1978). However, the risk of developing a second breast cancer related to the radiation treatment of the first primary breast was only suggested by Hankey *et al.* (1983), and a relation could not be confirmed by others (Basco *et al.*, 1985; McCredie *et al.*, 1975; Harvey & Brinton, 1985). Only the two studies based on data from the Connecticut Tumor Registry (Hankey *et al.*, 1983; Harvey & Brinton, 1985) contained sufficient numbers to detect a small radiation effect. In one study, an effect of radio-therapy was only observed among cases treated in recent years (Hankey *et al.*, 1983). In the other, the overall risk of contralateral breast cancer was higher among irradiated than in not irradiated (Harvey & Brinton, 1985) but the RR decreased with time since diagnosis of the first breast cancer, which is not in agreement with a radiation effect. Both studies, however, were based on data routinely collected by the Connecticut Tumor Registry, and the validity of the information, on treatment was not evaluated.

The present population based study summarizes more than 38 years experience of contralateral breast cancer among 56,237 women with breast cancer in Denmark. The risk is investigated in

Correspondence: H.H. Storm.
Received 21 January 1986; and in revised form, 9 June 1986.

© The Macmillan Press Ltd., 1986

relation to age, stage and treatment of disease at the primary breast cancer diagnosis, which has been recorded in a uniform manner since 1943. The possible effect of radiation treatment on the subsequent breast cancer risk is evaluated in this large cohort of women followed for a sufficiently long time to take account of latency between exposure and possible cancer development.

### Materials and methods

The Danish Cancer Registry is a population based, national cancer registry founded in 1942 and incidence data are available since 1943. Reports are received on newly diagnosed cancers by agreement between the National Board of Health, the Danish Medical Association and the Cancer Registry. Multiple reports are received for each case. A fee has been paid for each notification, and although reporting is voluntary several validation studies have shown that the registration may be regarded as complete (Clemmesen, 1965; Østerlind & Jensen, 1985), and similar to compulsory cancer registration schemes where completeness has been assessed (Lund, 1981). The registry is tumour based and uses an extended version of the seventh revision of the International Classification of Diseases (World Health Organization, 1957) for classification of cancers as described in detail by Jensen *et al.* (1985).

Cancers of paired organs are recorded as one tumour, with the date of the first cancer taken as date of diagnosis. However, primary bilateral breast cancers (i.e. simultaneous development within one month of left and right sided breast cancer) and contralateral breast cancer, defined as non-simultaneous independent cancer development in both breasts, are indicated by a special code in the tumour record. The decision of the presence of two independent breast cancers is left to the notifying physician and pathologist (Jensen *et al.*, 1985), who in general comply with the rules of Robbins & Berg (1964) (Nielsen *et al.*, 1986). Whenever the registry is in doubt as to the tumour diagnosis reported, the notifying physician is contacted.

### *Validation of cancer registry information*

Information on stage and treatment is recorded routinely. For all incident cases reported until 1977 no distinction was made in the coding, no radiotherapy and no information reported.

As part of this study the coding of information on treatment was therefore evaluated. Among the 2,588 patients with more than one breast cancer, 88.9% (1,451) of the ones classified as irradiated were reported as such, while 8.5% should have been classified as treatment unknown and 2.6% as not irradiated. Among those 1,137 patients classified as not irradiated (incl. treatment unknown), 35.0% should be classified as treatment unknown, 37.0% as irradiated and 28.0% as truly not irradiated.

A further validity check was performed by linking the registry information to the detailed information of treatment obtained in the Danish part of a study on second primary breast cancer following cancer of the cervix uteri (Day & Boice, 1983). Out of 243 second primary breast cancer cases, 26 (10.7%) had never been reported to the registry. Among the remaining 217 cases, 88.9% (112) of those coded as irradiated were in fact irradiated for breast cancer, whereas 45% (41) of those classified as not irradiated (i.e. not irradiated or radiation unknown) were in fact irradiated. In conclusion then some 90% of breast cancers classified as 'irradiated' are truly so, whereas some 40% of those 'non-irradiated' or 'irradiation unknown' were infact irradiated and this obviously limits the value of this latter group for comparison.

### *Cohort of breast cancer patients*

A total of 56,809 notified breast cancer cases diagnosed between January 1, 1943 and December 31, 1980 survived for more than 1 month. Altogether 2,588 or some 4.5% of the records were marked as either bilateral or contralateral breast cancer cases. The records of these cases were identified in the registry card file, and they were recoded with respect to date of diagnosis of second primary breast cancer and laterality, while ICD-O topography and morphology (WHO, 1976) were recoded for both the first and second primary breast cancers. The treatment was taken as coded routinely by the registry. The unilateral breast cancers were found to be correctly coded as such by recoding a random sample of 493 breast cancer cases from the years 1943 to 1980.

There was histological confirmation for 95% of the first primary breast cancers (Ewertz & Mouridsen, 1985) and 84% of the second breast cancers. Some 44% of the first and second breast cancers had different morphologies to the 4-digit level of the ICD-O.

Altogether 572 bilateral breast cancer patients were excluded from the study cohort. On recoding, 176 women were not counted as having developed a new cancer, as they were incorrectly coded (122 cases) or the contralateral 'cancer' was a benign or a carcinoma *in situ* lesion (54 cases). Altogether 56,237 women thus remained in the cohort for analysis. Excluding coding errors and simultaneous breast cancer cases, 1840 contralateral breast cancers were observed and included in the study.

Based on the age-specific incidence of contralateral breast cancer during the period 1943–80 the probability of developing contralateral breast cancer before the age 75 years was estimated as the cumulative incidence (Day, 1982). For comparison, the expected probability for breast cancer in the general population was calculated.

*Follow-up and analysis*

Follow-up information necessary for analysis has been recorded routinely by the Danish Cancer Registry, as the total register has been matched annually with all deaths since 1943, and dates of deaths recorded. Women for whom no date of death was present were considered to be alive. Emigration may be regarded as negligible among these patients as only 0.5% of the entire Danish population emigrated in the years 1941–80 (Danmarks Statistik, 1985). Person-years at risk were calculated from the time of diagnosis of the first breast cancer until the diagnosis of a cancer in the contralateral breast, date of death, December 31, 1980 or age 100 years (Monson, 1974), whichever occurred first. Expected numbers were computed by multiplying the person-years at risk in 5 year age and calendar periods with the corresponding incidence rates for breast cancer in all of Denmark, thereby adjusting indirectly for age and calendar time effects when summed over the cells (Monson, 1974). Relative risks (RR) were obtained by dividing the observed by the expected number of cases, and 95% confidence limits of the RR were calculated, assuming a Poisson distribution (Rothman & Boice, 1982). Tests for trend were conducted as described by Armitage (1955) using programs for a programmable calculator (Rothman & Boice, 1982). Tests for difference between observed and expected ratios were carried out as described by Haldane (1955).

The 56,237 primary breast cancer patients under study accrued 345,573.5 person-years of observation with an average age at diagnosis of 60.2 years and an average follow-up period of 6.2 years (Table I). Some 69% of the patients were recorded as receiving radiotherapy as part of the initial treatment. The highest proportion irradiated was among patients with regional metastasis at primary diagnosis (77%–88%) and lowest if the stage was unknown. Generally across all stages fewer patients aged 55 years or more at diagnosis were irradiated. For irradiated and non-irradiated women the mean ages within the various age-groups are similar except that patients not receiving radiation tend to be older than the irradiated women, if aged 55 or more years at primary breast cancer diagnosis. The patients that received radiation as primary treatment were on average followed 1.1 years longer than the ones that did not receive radiation, but this can be attributed to the longer follow-up of the women in the age-group 55+ years at diagnosis.

Eleven of the contralateral breast cancer cases were reported treated at first primary breast cancer with tamoxifen or cytotoxic drugs alone or in combination. These cases were diagnosed in the calendar period 1978–80 and are included in the analysis.

### Results

Table II shows that the overall RR of second primary breast cancer development is 2.8. The RR decreases from 5.5 if a woman is <45 years old at first breast cancer diagnosis to 2.1 if she is 55 years or more. A 60% significant difference (CI 95%, 40–80%) is observed between the RR's of age group <45 years and 45–54 years and between age group 45–54 years and 55+ years irrespective of treatment, as well as a significantly decreasing trend in RR is observed by increasing age ($P$ trend $<0.001$). The non-irradiated patients overall have a significantly higher RR for contralateral breast cancer than the irradiated patients.

The RR by treatment and years since first breast cancer is given in Table III. The overall relative risk is significantly increased in all time periods. A slight decrease in RR from 3.1 within the first 5 years of observation to 1.9, 25–29 years after primary diagnosis is observed, but the RR then increases among patients observed 30 or more years. The RR among irradiated patients is virtually stable during the first 20 years of observation (RR=2.1–2.8) with an indication of an increase (RR=4.2) 30 years or more after initial treatment. Among non irradiated patients on the contrary the RR are higher during the first 14 years of observation (RR=4.6–2.6), but declines to 1.6 15–19 years after and to insignificant levels of RR=1.5 20–29 years after initial treatment. As for the irradiated patients an increase in RR is observed after 30 years of observation.

If the first 9 years of observation are excluded, where a radiation effect would be less likely and the data less influenced by misclassification of metastasis, a significant 30% (CI 95%: 10–40%) difference between the irradiated (RR=2.6) and the non-irradiated groups (RR=2.0) emerges.

The RR of second primary breast cancer by age, stage and treatment of the first primary breast cancer excluding the first 4 years of observation is presented in Table IV. No change in the inverse relationship of contralateral breast cancer risk by age is observed, when stratifying for stage and treatment with irradiation. Among irradiated women the RR of second primary breast cancer increases with stage irrespective of age, with the

**Table I** Number of patients, average age at diagnosis of the primary breast cancer, average years of follow-up and number of person-years for the various cohorts of patients included in the analysis of contralateral breast cancer risk in Denmark 1943–80

| | *Number of patients Radiation* | | | | *Average age (years) Radiation* | | *Average follow-up (years) Radiation* | | *Number of person-years Radiation* | |
|---|---|---|---|---|---|---|---|---|---|---|
| | *Yes* | % | *No*[a] | % | *Yes* | *No*[a] | *Yes* | *No*[a] | *Yes* | *No*[a] |
| Extent of disease age group | | | | | | | | | | |
| Localized | | | | | | | | | | |
| <45 years | 3,039 | 79.3 | 793 | 20.7 | 39.7 | 39.5 | 7.8 | 6.8 | 23,808.9 | 5,352.1 |
| 45–54 years | 5,354 | 80.9 | 1,267 | 19.1 | 50.0 | 49.9 | 8.0 | 7.0 | 43,044.4 | 8,666.9 |
| >55 years | 2,306 | 70.0 | 5,263 | 30.0 | 67.1 | 72.8 | 6.5 | 4.5 | 79,806.7 | 23,037.9 |
| Regional | | | | | | | | | | |
| <45 years | 971 | 87.5 | 139 | 12.5 | 39.1 | 39.6 | 4.5 | 5.3 | 4,323.6 | 735.8 |
| 45–54 years | 1,620 | 87.8 | 226 | 12.2 | 50.1 | 50.5 | 4.9 | 4.9 | 7,978.9 | 1,042.0 |
| >55 years | 4,389 | 76.7 | 1,335 | 23.3 | 67.7 | 74.1 | 3.6 | 2.7 | 15,900.4 | 3,612.9 |
| Distant | | | | | | | | | | |
| <45 years | 172 | 69.6 | 75 | 30.4 | 40.0 | 39.7 | 4.1 | 3.4 | 700.7 | 263.4 |
| 45–54 years | 360 | 67.9 | 170 | 32.1 | 50.3 | 50.9 | 3.3 | 2.4 | 1,168.6 | 426.9 |
| >55 years | 1,037 | 46.4 | 1,198 | 53.6 | 67.5 | 72.0 | 2.3 | 1.4 | 2,331.9 | 1,580.4 |
| Unknown | | | | | | | | | | |
| <45 years | 1,762 | 63.3 | 1,021 | 36.7 | 39.5 | 39.5 | 11.2 | 12.3 | 19,743.0 | 12,526.0 |
| 45–54 years | 2,422 | 60.5 | 1,582 | 39.5 | 49.9 | 49.8 | 9.7 | 10.5 | 23,545.5 | 16,312.8 |
| >55 years | 5,147 | 52.9 | 4,589 | 47.1 | 67.1 | 69.3 | 5.4 | 4.8 | 27,527.0 | 22,394.3 |
| All Stages | | | | | | | | | | |
| <45 years | 5,944 | 74.6 | 2,028 | 25.4 | 39.6 | 39.5 | 8.2 | 9.3 | 48,568.5 | 18,878.6 |
| 45–54 years | 9,756 | 75.0 | 3,245 | 25.0 | 50.0 | 50.0 | 7.8 | 8.2 | 75.719.3 | 26,454.8 |
| >55 years | 22,879 | 64.9 | 12,385 | 35.1 | 67.2 | 71.6 | 5.5 | 4.1 | 125,509.8 | 50,647.1 |
| All | 38,579 | 68.6 | 17,658 | 31.4 | 58.5 | 63.9 | 6.5 | 5.4 | 249,630.9 | 95,942.6 |

[a]No radiation includes radiation unknown.

**Table II** Relative risk of cancer of the contralateral breast among women with breast cancer in Denmark 1943–80 in relation to age at first breast cancer and initial treatment

| *Age of diagnosis (years)* | *Irradiated* | | | | *Non-irradiated*[a] | | | | *All* | | | |
|---|---|---|---|---|---|---|---|---|---|---|---|---|
| | O | E | RR | 95% CI | O | E | RR | 95% CI | O | E | RR | 95% CI |
| <45 | 269 | 57.5 | 4.7 | (4.1–5.3) | 167 | 22.5 | 7.4 | (6.3–8.6) | 436 | 80.0 | 5.5 | (5.0–6.0) |
| 45–54 | 359 | 120.3 | 3.0 | (2.7–3.3) | 188 | 41.4 | 4.5 | (3.9–5.2) | 547 | 161.7 | 3.4 | (3.1–3.7) |
| >55 | 544 | 285.1 | 1.9 | (1.8–2.1) | 313 | 121.7 | 2.6 | (2.3–2.9) | 857 | 406.8 | 2.1 | (2.0–2.3) |
| Total | 1,172 | 462.9 | 2.5 | (2.4–2.7) | 668 | 185.6 | 3.6 | (3.3–3.9) | 1,840 | 648.5 | 2.8 | (2.7–3.0) |

[a]No radiation includes radiation unknown.

**Table III** Observed numbers and RR of contralateral breast cancer by treatment and time since first breast cancer in Denmark 1943–80

| *Years since first breast cancer* | *Irradiation* | | | *No irradiation*[a] | | | *All* | | |
|---|---|---|---|---|---|---|---|---|---|
| | O | RR | CI 95% | O | RR | CI 95% | O | RR | CI 95% |
| 0–4 | 563 | 2.5 | 2.3–2.7 | 414 | 4.6 | 4.1–5.0 | 977 | 3.1 | 2.9–3.3 |
| 5–9 | 282 | 2.5 | 2.2–2.8 | 136 | 3.8 | 3.1–4.4 | 418 | 2.8 | 2.5–3.1 |
| 10–14 | 153 | 2.5 | 2.1–2.9 | 59 | 2.6 | 2.0–3.3 | 212 | 2.5 | 2.2–2.9 |
| 15–19 | 87 | 2.6 | 2.1–3.2 | 26 | 1.6 | 1.1–2.4 | 113 | 2.3 | 1.9–2.7 |
| 20–24 | 52 | 2.8 | 2.1–3.7 | 17 | 1.5 | 0.9–2.4 | 69 | 2.3 | 1.8–2.9 |
| 25–29 | 19 | 2.1 | 1.3–3.3 | 9 | 1.5 | 0.7–2.9 | 28 | 1.9 | 1.2–2.7 |
| 30+ | 16 | 4.2 | 2.3–6.7 | 7 | 3.3 | 1.3–6.9 | 23 | 3.8 | 2.4–5.8 |
| Total excluding 0–9 years | 327 | 2.6 | 2.3–2.9 | 118 | 2.0 | 1.7–2.4 | 445 | 2.4 | 2.2–2.6 |

[a]No irradiation includes radiation unknown.

**Table IV** Relative risk of contralateral breast cancer among women with breast cancer in Denmark 1943–80 by age at diagnosis, extent of disease and radiation treatment to the first breast cancer (observed and expected number of cases during first 5 years of follow-up excluded)

| *Stage* | | *Irradiated* | | | *Not irradiated*[a] | | |
|---|---|---|---|---|---|---|---|
| | | <45[b] years | 45–54 years | 55+ years | <45 years | 45–54 years | 55+ years |
| *Local* | O | 84 | 129 | 192 | 16 | 17 | 22 |
| | E | 16.6 | 36.8 | 82.2 | 4.2 | 8.0 | 22.6 |
| | RR | 5.1 | 3.5 | 2.3 | 3.8 | 2.1 | 1.0 |
| | 95% CI | 4.0–6.3 | 2.9–4.2 | 2.0–2.7 | 2.2–6.2 | 1.2–3.4 | 0.6–1.5 |
| *Regional metastases* | O | 15 | 24 | 26 | 0 | 3 | 2 |
| | E | 2.5 | 5.7 | 11.4 | 0.5 | 0.7 | 2.2 |
| | RR | 6.0 | 4.2 | 2.3 | 0.0 | 4.3 | 0.9 |
| | 95% CI | 3.4–9.9 | 2.7–6.3 | 1.5–3.3 | 0.0–7.3 | 0.9–12.5 | 0.1–3.3 |
| *Distant metastases* | O | 6 | 3 | 7 | 0 | 2 | 2 |
| | E | 0.3 | 0.6 | 1.2 | 0.2 | 0.2 | 0.7 |
| | RR | 20.0 | 5.0 | 5.8 | 0.0 | 10.0 | 2.9 |
| | 95% CI | 7.3–43.5 | 1.0–14.6 | 2.3–12.0 | 0.0–18.3 | 1.1–36.1 | 0.3–10.3 |
| *Unknown* | O | 29 | 52 | 42 | 47 | 52 | 91 |
| | E | 20.1 | 28.1 | 32.9 | 12.7 | 19.2 | 40.9 |
| | RR | 1.4 | 1.9 | 1.3 | 3.7 | 2.7 | 2.2 |
| | 95% CI | 1.0–2.1 | 1.4–2.4 | 0.9–1.7 | 2.7–4.9 | 2.0–3.6 | 1.8–2.7 |
| *All* | O | 134 | 208 | 267 | 63 | 74 | 116 |
| | E | 39.5 | 71.3 | 127.7 | 17.5 | 28.2 | 49.3 |
| | RR | 3.4 | 2.9 | 2.1 | 3.6 | 2.6 | 2.4 |
| | 95% CI | 2.8–4.0 | 2.5–3.3 | 1.8–2.4 | 2.8–4.6 | 2.1–3.3 | 1.9–2.8 |

[a]Not irradiated includes radiation unknown; [b]age at diagnosis of first breast cancer.

lowest values following a localized primary breast cancer and the highest values if the first primary breast cancer had distant metastasis. Numbers are too small to evaluate the influence of stage in the non-irradiated group.

For the large group of women with localized disease there is a significant higher risk among irradiated women followed for 5 or more years irrespective of age (405 observed, 135.6 expected, RR=3.0; CI 95%; 2.7–3.3) than among non-irradiated (55 observed, 34.8 expected RR=1.6; CI 95%; 1.2–2.1) (Table IV). However taking age at primary breast cancer into consideration, the observed number of cases is small and confidence intervals thus wide in particular for the non-irradiated group (Figure 1). No major difference in RR is seen between irradiated and non-irradiated premenopausal (<45 years) and menopausal (45–54 years) women during the first 19 years of follow-up. By contrast a slightly increasing risk, significantly different between irradiated and not irradiated postmenopausal women (55+ years), is observed 10–14 years after first breast cancer. The difference remains throughout the follow-up period, however numbers in the non-irradiated group are too small to make the difference significant.

### Discussion

The RR or incidence of contralateral breast cancer has previously been assessed, mostly in hospital based studies (Haagensen, 1971; Robbins & Berg, 1964; Schottenfeld & Berg, 1971; McCredie *et al.*, 1975; Hislop *et al.*, 1984; Chaudery *et al.*, 1984).

**Figure 2** Cumulative risk of developing breast cancer before the age of 75 years for women with and without breast cancer at different ages. Probability of breast cancer (---) and of contralateral breast cancer (——).

The risk estimates of these often smaller studies are in concordance with those obtained in population based studies (Prior & Waterhouse, 1978; Hankey *et al.*, 1983; Harvey & Brinton, 1985) with an incidence of 3.8–7.1 per 1000 women-years. With 345,000 women-years of observation the present study is the largest population based investigation of contralateral breast cancer risk till now. The RR including more than 30 years of observation is 2.8, which corresponds to a crude incidence of 5.3 per 1000 women-years.

The RR of 2.8 found in our study is similar to the ones seen in Connecticut RR=3.0–3.2 (Hankey *et al.*, 1983; Harvey & Brinton, 1985) and Birmingham RR=2.4 (Prior & Waterhouse, 1978).

**Figure 1** Relative risk of contralateral breast cancer, by age, treatment and time since a localized primary breast cancer. Irradiated (---); non-irradiated (——).

The similarities of the risk estimates from different studies are noteworthy considering the various definitions used for second primary breast cancer. Many authors (Prior & Waterhouse, 1978; Chaudery *et al.*, 1984; Basco *et al.*, 1985; Nielsen *et al.*, 1986) attempt to exclude metastatic spread of the first breast cancer accepting the second by applying the rules of Robbins & Berg (1964) which either require a difference in morphology between the two tumours or presence of contiguous *in situ* lesions associated with the second primary invasive breast cancer. Whether these rules have been applied by the reporting physicians in Denmark is unknown. Prior & Waterhouse (1978) obtained conservative estimates by excluding all primaries with known metastases, as the present study and the Connecticut studies (Harvey & Brinton, 1985; Hankey *et al.*, 1983) may have done, by excluding all simultaneous cases (developing within the first months of observation, one and two respectively). To minimize the influence of misclassified metastasis we excluded the numbers from the first 5 years of observation from some of the totals, thereby excluding 50% of all contralateral breast cancers and lowering the overall risk from 2.8 to 2.4. However, a recent Danish study on the pathology of second primary breast cancer (Nielsen *et al.*, 1986) indicates that approximately 50% of all second primary breast cancer does occur within this time interval

The RR for contralateral breast cancer seen in this investigation (Table II) is significantly increased for both premenopausal (age $<45$ years), menopausal (age 45–54 years) and postmenopausal (age 55+ years) women, but decreases with increasing age at first breast cancer. This is in agreement with previous observations (Hankey *et al.*, 1983; Prior & Waterhouse, 1978). A significant decreasing RR with increasing age ($P<0.05$) is present (Tables II and IV), and seen during the years of follow-up irrespective of treatment (Figure 1). The trend 15 and more years after diagnosis is difficult to evaluate, as the findings are influenced by small numbers. Considering only pre- and post-menopausal women (age $<45$ years, age 55+ years) all stages and treatments combined, a difference in RR between these two age groups is present in all time intervals since primary breast cancer, but this is not significant after 15 years of follow-up.

It has been suggested that young breast cancer patients represent a genetically predisposed group (Anderson, 1977) and this has been offered as an explanation for the particular increased RR of contralateral breast cancer among young women (Anderson, 1977; Prior & Waterhouse, 1978; Fraumeni, 1977). However, a case control study among patients with contralateral breast cancer in Sweden showed no increased RR with a positive family history (Adami *et al.*, 1981). Another explanation for the high risk observed may be that young breast cancer patients represent a group more exposed to exogenous risk factors than older patients.

The stage of the first primary breast cancer (Table IV) is directly related to the risk of second primary breast cancer as women with a localized first breast cancer have the lowest RR. Stage is, however, a weak risk factor and it is associated with a significant risk elevation between the localized and the distant metastatic primary breast cancers only among the young irradiated women. A similar influence of stage at first primary breast cancer was observed by Hankey *et al.* (1983), and attributed to possible genetic predisposition if node-involvement (metastatic spread) was present at initial diagnosis.

Radiation is a known risk factor for breast cancer (MacMahon *et al.*, 1973; Cole, 1980), and there is evidence of a latent period of at least 5–15 years between exposure and breast cancer development (Baral *et al.*, 1977; Boice & Monson, 1977; Land, 1980*a*, Land *et al.*, 1980*b*). The risk associated with radiation continues throughout life, based on data with exposure to radiation before (Tokunaga *et al.*, 1982) and after puberty and before menopause. Uncertainties exist about breast cancer risk from exposures after menopause (Land, 1980). In the present study irradiated women followed for 10 or more years have a RR of 2.6, which is significantly higher than among the non-irradiated (RR 2.0) (Table III). This approximately 30% higher risk among the irradiated women may be regarded as conservative, as some 40% of the 'non-irradiated' women in fact received radiotherapy, which may have elevated the level of risk in the comparison group. Misclassification of radiation exposure may also be larger for contralateral breast cancer patients than for single breast cancer cases, as only 56% of the first primary breast cancers among contralateral cases were classified as irradiated, contrary to 69% of the single breast cancer patients. The RR observed may thus be even more conservative than what would be attributable to a proportional shift in exposure category. This is further supported by our experience from a study of second cancer following cancer of the cervix uteri where the recorded elevation of RR among the irradiated women would be conservative due to misclassification of treatment, especially among patients with more than one cancer (Storm & Boice, 1985*a*; Storm *et al.*, 1985*b*). The RR is thus likely to be underestimated in the irradiated group and overestimated in the non-irradiated group.

The RR of contralateral breast cancer is most difficult to evaluate in women with metastasis at first breast cancer diagnosis. When considering only the largest group of patients with a localized primary breast cancer, we find consistent higher RR's in all age-groups irradiated than among non-irradiated women surviving 20 years or more (Figure 1). However, the difference is only significant for ages 45–54 years at initial diagnosis or when all ages are combined but the difference in RR between the two treatment groups is likely to be underestimated as mentioned earlier. The present findings are thus in concordance with a possible influence of radiation on breast cancer risk of long term surviving women with breast cancer in particular at premenopausal and menopausal age. The increase in RR for women irradiated below age 45 years, between 45 and 54 years and after 55 years of age is expressed at ages above 60 years, indicating an interplay of both an age effect and a radiation effect, among long term survivors.

The contralateral breast may receive between 50–200 cGy by the treatment of the primary breast cancer (Benedick *et al.*, 1985). With current risk estimates for radiogenic breast cancer (6.6 cases/rad/$10^6$ women-years) (Land *et al.*, 1980) this would give rise to 82–329 breast cancers or account for 11–46% of the observed excess among the irradiated in our study. Risk estimates for radiation-induced breast cancer is based on sufficient data for women aged below 40 at exposure (Land *et al.*, 1980), whereas data on women aged 50 or more years at exposure are less reliable due to small numbers (Tokunaga *et al.*, 1979). Baral *et al.* (1977) reported a decreasing excess risk per rad with increasing age at exposure (although dose was highly correlated with age at treatment), a finding supported by animal data quoted in the report from the committee on the biological effects of ionizing radiations (BEIR) (1980). The average age at exposure in our study was 60 years and the number of cancers attributed to radiation may be smaller than estimated.

A recent study by Basco *et al.* (1985) found no evidence of radiation-induced carcinogenesis among 194 contralateral breast cancer patients with an average exposure of 138–321 cGy to the contralateral breast. However, the study population was small and included only 44 contralateral breast cancer cases observed for a sufficient time period (>10 years) after exposure to be able to observe any effect of radiation. In addition some 50% of these 44 patients are suspected to be above the age of 50 years at exposure, where a radiation effect is likely to be less pronounced (Land *et al.*, 1980).

In summary then the present study confirms that women with breast cancer are at increased risk of developing a new clinically manifest primary cancer of the contralateral breast. The risk is influenced by the age at diagnosis, the stage of the disease when first diagnosed, and possibly by radiotherapy to the first breast cancer. In addition to the possible leads from these observations it is important from a clinical aspect that some 25% of women with breast cancer below the age of 45 years develop a contralateral breast cancer if surviving to the age of 75 years, while 6% of women aged 55 years or more at diagnosis are likely to get a new breast cancer (Figure 2). These probabilities are in agreement with the observations reported by Foote & Steward (1945) and Robbins & Berg (1964). As the increased RR persists it is important to follow especially young breast cancer patients clinically with respect to new breast cancer development for life.

Clarification of the role of radiation in breast cancer development is important, especially considering treatment of primary localized breast cancer with partial mastectomy/lumpectomy, axillary dissection and adjuvant radiotherapy. The present study is supportive of breast cancer induction as a long term side-effect of radiation. The role of radiation in the development of cancer of the contralateral breast can only be completely assessed in a well designed study among long term survivors with individual radiation dosimet[illegible] performed. Coordinated case control studies in Denmark and Connecticut that address this issue are under way. Studies of other exogenous factors related to both the primary and the contralateral breast cancer are also needed, for the identification of high risk groups.

The authors wish to thank Niels Christensen for data processing assistance, Aase Larsen for preparing the graphs and Helle Nielsen for preparing manuscript and tables.

Part of this work was supported by National Institutes of Health, National Cancer Institute, USA, purchase order no. 263-MD-319265.

### References

ADAMI, H., HANSEN, J., JUNG, B. & RIMSTEN, A. (1981). Characteristics of family breast cancer in Sweden. Absence of relation to age and unilateral versus bilateral disease. *Cancer*, **48**, 1688.

ANDERSON, D.E. (1977). Breast cancer in families. *Cancer*, **40**, 1855.

ARMITAGE, P. (1955). Tests for linear trends in proportions and frequencies. *Biometries*, **11**, 375.

BARAL, E., LARSSON, L.E. & MATTSON, B. (1977). Breast cancer following irradiation of the breast. *Cancer*, **40**, 2905.

BASCO, V.E., COLDMAN, A.J., ELWOOD, J.M. & YOUNG, M.E.J. (1985). Radiation dose and second breast cancer. *Br. J. Cancer*, **52**, 319.

BENEDICK, A.F., ROBERTSON, P.L. & LICHTER, A.S. (1985). Dose to the contralateral breast due to primary breast irradiation. *Int. J. Radiation Oncology Biol. Phys.*, **11**, 485.

BOICE, J.D. Jr. & MONSON, R.R. (1977). Breast cancer after repeated fluoroscopic examinations of the chest. *J. Natl Cancer Inst.*, **59**, 823.

CHAUDERY, M.A., MILLIS, R.R., HOSKINS, E.D.L. & 4 others. (1984). Bilateral primary breast cancer: a prospective study of disease incidence. *Br. J. Surg.*, **71**, 711.

CLEMMESEN, J. (1965). Statistical studies in the aetiology of malignant neoplasms. Volume 1. *Acta path. microbiol. Scand.*, Suppl. **174**, 52.

COLE, P. (1980). Major aspects of the epidemiology of breast cancer. *Cancer*, **46**, 865.

COMMITTEE ON THE BIOLOGICAL EFFECTS OF IONIZING RADIATIONS (1980). National Research Council, National Academy of Sciences. The effects on populations of exposure to low levels of ionizing radiation. P. 168, National Academy Press: Washington DC.

DANISH CANCER REGISTRY (1983). Cancer incidence in Denmark 1978, 1979 and 1980. Danish Cancer Society: Copenhagen.

DANMARKS STATISTIK (1985). Statistical yearbook, **89**, 11. Copenhagen.

DAY, N.E. (1982). Cumulative rate and cumulative risk. In *Cancer incidence in five Continents*, Waterhouse *et al.* (eds) p. 668. International Agency for Research on Cancer: Lyon.

DAY, N.E. & BOICE, J.D. Jr (ed). (1983). Introductory chapter. In *Second cancer in relation to radiation treatment for cervical cancer*. P. 11, IARC Scientific publication No. 52: Lyon.

EWERTZ, M. & JENSEN, O.M. (1981). Breast Cancer in Denmark 1943–76. *Ugeskr. Laeger.*, **143**, 2758. (in Danish).

EWERTZ, M. & MOURIDSEN, H.T. (1985). Second cancer following cancer of the breast in Denmark 1943–80. *Natl. Cancer Inst. Monogr.*, **68**, 325.

FISCHER, B., BAUER, M., MARGOLESE, R. & 16 others. (1985). Five-year results of a randomized clinical trial comparing total mastectomy and segmental mastectomy with or without radiation in the treatment of breast cancer. *N. Engl. J. Med.*, **312**, 665.

FOOTE, F.E. & STEWART, F.W. (1945). Comparative studies of cancerous vs. noncancerous breasts – I and II. *Ann. Surg.*, **191**, 6 (part I), 197 (part II).

FRAUMENI, J.F. Jr. (1977). Clinical patterns of familial cancer. In *Progress in Cancer research and therapy* **3**. Genetics of Human Cancer, Mulvihill *et al.* (eds) p. 223. Raven Press: New York.

HAAGENSEN, C.D. (1971). *Disease of the Breast*. p. 449. Saunders: Philadelphia.

HALDANE, J.B.S. (1955–56). The estimation and significance of the logarithm of a ratio of frequencies. *Ann. Human Genet.*, **20**, 309.

HANKEY, B.F., CURTIS, R.E., NAUGHTON, M.D., BOICE, J.D. Jr. & FLANNERY, J.T. (1983). A retrospective cohort analysis of second breast cancer risk for primary breast cancer patients with an assessment of the effect of radiation therapy. *J. Natl Cancer Inst.*, **70**, 797.

HARVEY, E.B. & BRINTON, L.A. (1985). Second cancer following cancer of the breast in Connecticut 1935–82. *Natl. Cancer Inst. Monogr.*, **68**, 99.

HISLOP, T.G., ELWOOD, J.M., COLDMAN, A.J., SPINELLI, J.J., WORTH, A.J. & ELLISON, L.G. (1984). Second primary cancers of the breast: Incidence and risk factors. *Br. J. Cancer*, **48**, 79.

JENSEN, O.M., STORM, H.H. & JENSEN, H.S. (1985). Cancer registration in Denmark and the study of multiple primary cancers 1943–80. *Natl. Cancer Inst. Monogr.*, **68**, 245.

LAND, C.E. (1980*a*). Low-dose radiation. A cause of breast cancer? *Cancer*, **46**, 868.

LAND, C.E., BOICE, J.D. Jr., SHORE, R.E., NORMAN, J.E. & TOKUNAGA, M. (1980*b*). Breast cancer risk from low-dose exposures to ionizing radiation: Results of parallel analysis of three exposed populations of women. *J. Natl Cancer Inst.*, **65**, 353.

LEVITT, S.H. (1980). The role of radiation therapy in the treatment of breast cancer: The use and abuse of clinical trials, statistics, and unproven hypotheses. *Int. J. Radiat. Oncol. Biol. Phys.*, **6**, 791.

LUND, E. (1981). Pilot study for the evaluation of completeness of reporting to the cancer registry. In *Incidence of Cancer in Norway 1978*. P. 11. The Cancer Registry of Norway.

McCREDIE, J.A., INCH, W.R. & ALDERSON, M. (1975). Consecutive primary carcinomas of the breast. *Cancer*, **35**, 1472.

MACMAHON, B., COLE, P. & BROWN, J. (1973). Etiology of human breast cancer. A review. *J. Natl Cancer Inst.*, **50**, 21.

MOLE, R.H. (1978). The sensitivity of the human breast to cancer induction by ionizing radiation. *Br. J. Radiol.*, **51**, 401.

MONSON, R.R. (1974). Analysis of relative survival and proportional mortality. *Comput. Biomed. Res.*, **7**, 325.

NIELSEN, M., CHRISTENSEN, L. & ANDERSEN, J. (1986). Contralateral cancerous breast lesions in women with clinical invasive breast carcinoma. *Cancer*, **57**, 897.

PRIOR, P. & WATERHOUSE, J.A.H. (1978). Incidence of bilateral tumours in a population based series of breast-cancer patients. I. Two approaches to an epidemiological analysis. *Br. J. Cancer.*, **43**, 615.

ROBBINS, G.F. & BERG, J.W. (1964). Bilateral primary breast cancers a prospective clinicopathological study. *Cancer*, **17**, 1501.

ROTHMAN, K.J. & BOICE, J.D. Jr. (1982). Epidemiologic analysis with a programmable calculator, Epidemiology Resources, Inc.: Boston.

SCHOTTENFELD, D. & BERG, J. (1971). Incidence of multiple primary cancer, IV. Cancer of the female breast and genital organs. *J. Natl Cancer Inst.*, **46**, 161.

STORM, H.H. & BOICE, J.D. Jr. (1985*a*). Leukemia after cervical cancer irradiation in Denmark. *Int. J. Epid.*, **14**, 363.

STORM, H.H., JENSEN, O.M., EWERTZ, M. & 4 others. (1985*b*). Summary: • Multiple primary cancers in Denmark, 1943–80. *Natl. Cancer Inst. Monogr.*, **68**, 411.

TOKUNAGA, M., LAND, C.E., YAMAMOTO, T. & 4 others. (1982). Breast cancer in Japanese a-bomb survivors. *Lancet*, **ii**, 924.

TOKUNAGA, M., NORMAN, J.E. Jr., ASANO, M. & 4 others. (1979). Malignant breast tumors among atomic bomb survivors, Hiroshima and Nagasaki, 1950–74. *J. Natl Cancer Inst.*, **62**, 1347.

VERONESI, U., SACCOZZI, R., DEL VECCHIO, M. & 12 others. (1981). Comparing radical mastectomy with quadrantectomy, axillary dissection radiotherapy in patients with small cancer of the breast. *N. Engl. J. Med.*, **305**, 6.

WATERHOUSE, J.A.H., MUIR, C.S., SHANMUGARATNAM, K. & POWELL, J. (ed). (1982). Cancer incidence in Five Continents. **IV**, 671, IARC. Sci. Publ. No. 42, IARC: Lyon.

WHO (1976). International Classification of Diseases for Oncology, Geneva.

WHO (1957). International Classification of Diseases, 1955. Revision, Geneva.

ØSTERLIND, A. & JENSEN, O.M. (1985). Evaluation of registration of cancer cases in 1977. Preliminary evaluation of registration of cancer cases by the Cancer Registry and National Patient Registry. *Ugeskr. Laeg.*, **147**, 2483. (in Danish).